# Security of six-state quantum key distribution protocol with threshold detectors

**DOI:** 10.1038/srep30044

**Published:** 2016-07-22

**Authors:** Go Kato, Kiyoshi Tamaki

**Affiliations:** 1 NTT Communication Science Laboratories, NTT Corporation 3-1, Morinosato Wakamiya Atsugi-Shi, Kanagawa, 243-0198, Japan; 2 NTT Basic Research Laboratories, NTT Corporation, 3-1, Morinosato Wakamiya Atsugi-Shi, Kanagawa, 243-0198, Japan

## Abstract

The security of quantum key distribution (QKD) is established by a security proof, and the security proof puts some assumptions on the devices consisting of a QKD system. Among such assumptions, security proofs of the six-state protocol assume the use of photon number resolving (PNR) detector, and as a result the bit error rate threshold for secure key generation for the six-state protocol is higher than that for the BB84 protocol. Unfortunately, however, this type of detector is demanding in terms of technological level compared to the standard threshold detector, and removing the necessity of such a detector enhances the feasibility of the implementation of the six-state protocol. Here, we develop the security proof for the six-state protocol and show that we can use the threshold detector for the six-state protocol. Importantly, the bit error rate threshold for the key generation for the six-state protocol (12.611%) remains almost the same as the one (12.619%) that is derived from the existing security proofs assuming the use of PNR detectors. This clearly demonstrates feasibility of the six-state protocol with practical devices.

Quantum key distribution (QKD) allows legitimated users to securely communicate, and the security of QKD protocols has been well studied so far^1^. In the security proof, for simplicity of the analysis, it is quite common to put some assumptions on the devices that a QKD system employs. One of such assumptions is the use of the PNR detectors^2^, and this assumption is important for the six-state protocol. This is so because this allows us to exploit the properties of the qubit pairs, in particular the mutual information between the bit and the phase errors, and this boosts up the tolerable bit error rate for the key generation in the protocol. The problem is that the PNR detectors are demanding and it could be obstacle to implement the six-state protocol in practice. Therefore, it is important from the practical viewpoint to consider the security of the six-state protocol without the PNR detectors.

One of the ways to remove the assumption of the use of the PNR detectors is to implement detector decoy idea^3^, or estimation method via monitoring the double click event^4^, all of which require some modifications to QKD protocols. Another approach for the security proof of QKD with threshold detectors is to consider the so-called squash operator^5^ which squashes an optical mode down to a qubit state. This approach only requires to assign the double-click event (detectors “0” and “1” simultaneously click) to a random bit value^6^,^7^. The existence of the squash operator for BB84-type measurement has been proven^8^,^9^,^10^,^11^, i.e., the statistics of the outcomes of the BB84 measurement can be interpreted as if it stemmed from the BB84 measurement on qubits whatever optical signal Bob actually receives.

It might be likely that the squash operator exists for any measurement with two outcomes, including the measurement of the six-state protocol^12^,^13^, where we perform measurements along a basis, *Y* basis, in addition to *X* and *Z* bases in BB84. In the case of the qubit-based six-state protocol, the measurement along the extra basis, Y basis, lets us learn more about Eve’s information gain, resulting in a higher bit error rate threshold than that of BB84, which is a main advantage of the qubit-based six-state protocol over BB84. Unfortunately, it turns out that the squash operator for the six-state protocol is proven not to exist^10^, and it is unknown whether the advantage still holds with the use of threshold detectors.

Intuitively, sending more than one-photon is not useful for the eavesdropping since it may only increase the bit error rate, and it is hard to imagine that the advantage of the qubit-based six-state protocol suddenly vanishes once we lose information about which signal is a single-photon. In other words, to consider the security of the six-state protocol with threshold detectors is related to consider the robustness of a qubit-based QKD protocol even if there is no squash operator. This is indeed one of the essential features that any practical qubit-based QKD must possess, and this issue must be seriously taken into account for the design of a qubit-based QKD protocol.

In this work, we prove the robustness of the six-state protocol by showing the bit error rate threshold remains almost the same (12.611%) compared to the one of the qubit-based six-state protocol (12.619%). This result shows that sending multiple photons hardly helps Eve, which confirms the intuition mentioned above. The rate is clearly larger than the rate of BB84 with threshold detectors (11.002%)^8^,^9^,^10^, and this demonstrates the advantage of using two additional states in the practical situation. It is instructive to mention the difference of our work from a related work^14^. Our work exploit the existence of the squash operator for the six-state protocol up to two photons as well as the one for BB84 while the work in the paper^14^ demonstrates a universal idea applicable for many single-photon based protocols without relying on the existence of the squash operators. We remark that our work assumes the use of a single-photon as the information carrier, but we can trivially accommodate the use of an attenuated laser source by GLLP idea^5^.

## Results

### Brief description of the 6-state protocol

In this paper, we use 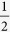-spin notation for the explanation since polarization state of a single-photon and the 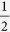-spin state are mathematically equivalent. In the six-state protocol, Alice first generates a random bit value *b* = −1, 1 and choose one basis *α* randomly out of three bases *X, Y*, and *Z*. Then, she sends over a quantum channel a qubit with state being 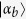 that is the eigen state of *α* basis of 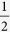-spin whose eigen value is *b*/2. Bob randomly chooses one basis out of the three bases, and he measures the spin along the chosen direction. Alice and Bob compare over a public channel the bases they used, and keep the bit value if the bases match, othrewise discard it. Alice and Bob repeat this step many times, and they apply bit error correction^15^ and privacy amplification^15^ to the resulting bit string (sifted key), and they share the key.

### Structure of the security proof

Our proof employs the security proof based on complementarity scenario proposed by Koashi^16^. In this proof, we consider two protocols, one is the actual protocol that Alice and Bob actually conduct, and the other one is a virtual protocol. Let us assume that Alice has a qubit state, which may be fictitious, and let the *Z* basis be Alice’s key generating qubit basis. The goal of the actual protocol is that Bob agrees on Alice’s bit values along the *Z* basis. On the other hand, the goal of the virtual protocol is to create an eigen state of an observable *X*, which is conjugate to the *Z* basis, with the help of Alice and Bob’s arbitrary quantum operations that commute with Alice’s key generating measurement. It is proven that if Alice and Bob are free to choose which protocol to execute after the actual classical and quantum communication and if they can accomplish its goal whichever choice they have made, then unconditionally secure key can be distilled.

In order to define Alice’s qubit in the six-state protocol, suppose that Alice first prepares a qubit pair in the state 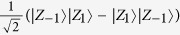 (We choose this singlet state to fully make use of its symmetry later. As a result, Alice and Bob’s bit values are anti-correlated with this state in the absence of Eve.), measures one of the qubit by the *X, Y*, or *Z* basis, and sends the other qubit to Bob. Since this process outputs the exactly the same state as the one of the actual protocol, we are allowed to work on this scenario without losing any generality. In the case that we consider the security of the key generated along the *Z* basis, and once Alice and Bob can generate |*X*_1_〉 state in Alice’s side in the virtual protocol then we are done since the agreement on the bit value in the actual protocol can be trivially made via classical error correction over a public channel (the syndrome is either encrypted^17^ or not^18^).

For the generation of |*X*_1_〉 state, an important quantity is the so-called phase error rate, which is the ratio that Bob’s estimation of Alice’s bit string in the *X*-basis results in erroneous, and if the estimation of the phase error rate is exponentially reliable then Alice can generate |*X*_1_〉 by random hashing along the *X* basis^17^,^18^,^19^. More precisely, the key generation rate *G*, assuming a perfect bit error correcting code, can be expressed as 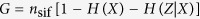. Here, *n*_sif_ is the empirical probability of having the sifted key, *H*(*X*) is Shannon entropy of the bit error, and *H*(*Z*|*X*) is Shannon entropy of the phase error conditional on the bit error pattern. In other words, *n*_sif_*H (X*) is the number of the hashing along the *Z* basis needed for the agreement of the bit values in the actual protocol and 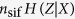 is the one along the *X* basis needed for the generation of the *X* basis eigen state in the virtual protocol^16^. Hence, the key for the improvement in the key generation rate is how to maximize *H (Z*|*X*). Thus, we are left with the estimation of the conditional entropy from the actually observed quantities.

For the estimation, we assume without loss of generality that states received by Bob are classical mixtures of photon number eigen states, and let *P*_*N*_ be the probability of receiving a state having *N* photons. We can make this assumption because introducing projection onto Fock space preceding Bob’s measurement does not change any measurement outcome. Important in the security proof is only the fact that we can *in principle* correct the phase errors^5^, and therefore we can consider the phase error rate separately for each of the photon number space. This means in particular that the key generation rate *G* becomes 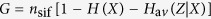. Here, 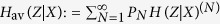 and 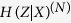 is the conditional Shannon entropy that is derived from *N*-photon detection event by Bob. However, we have no direct access to *P*_*N*_. Therefore, we have to assume the worst case scenario where Eve maximizes the induced phase error rate by classically mixing up each photon number state and sending them to Bob. As we will see later, it can be proven that states with photon number being greater than 3 induces too much bit errors and we can neglect those states for the analysis. Hence, we can concentrate only on *N* = 1, 2, 3 cases, and especially we want to derive the corresponding mutual information between the bit and phase errors.

### Eve’s information

To compute the mutual information, we introduce Bob’s qubit by employing the BB84 squash operator, and we have to estimate what statistics we would have obtained if we had performed the measurement along the 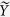 basis onto the resulting qubit (here, “tilde” means that this is about a qubit space and fictitous). In general, the actual Bob’s measurement along the *Y* basis does not coincide with the measurement along the 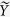 basis, however they do only when *N* = 1, 2 thanks to the existence of the squash operator for the six-state protocol^10^. This gives the same mutual information for *N* = 1, 2 as the one of the qubit-based six-state protocol. We note that to employ the BB84 squash operator, we have to randomly pick up two bases (for the explanation, we assume that we have chosen the *X* and *Z* bases) out of the three bases in the actual protocol. This random choice does not change the actual protocol at all. The reason is that we can always split the basis choice into two steps: the first one is the choice of two bases out of the three and then one basis is chosen from the two.

To analyze *N* = 3 case, we use the symmetry of the density operator. For simplicity of the analysis, we assume in the actual protocol that Alice and Bob perform joint random bit-flip operation to make the analysis simpler. As a result, we can estimate the mutual information. Finally, by mixing up the photon number state *N* = 1, 2, 3 based on the worst case scenario, we show that the bit error rate threshold for the six-state protocol with threshold detectors is 12.611%. In what follows, we give a detailed explanation of our security proof in which we take the asymptotic limit such that the number of the pulses is infinite and we neglect statistical fluctuations.

#### Analysis for *N* ≠ 3

Our goal is to maximize 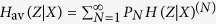 based on the worst scenario for *P*_*N*_. Imagine that we make a two-dimensional (2*D*) plot of 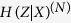 as a function of the bit error rate *e*_b_ for the *N*-photon state. The convex combination suggests that we have to consider a convex hull, each of whose extreme points corresponds to 
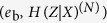
 in the 2*D* plane, and the 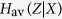 at the average bit error rate coincides with the observed error rate is in the convex hull. Thanks to the existence of the squash operator for the six-state protocol^10^, the plot of 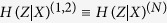 for *N* = 1, 2 is the same as the one of the qubit-based six-state protocol^12^,^13^, which is expressed as


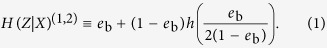


**Figure 1:**
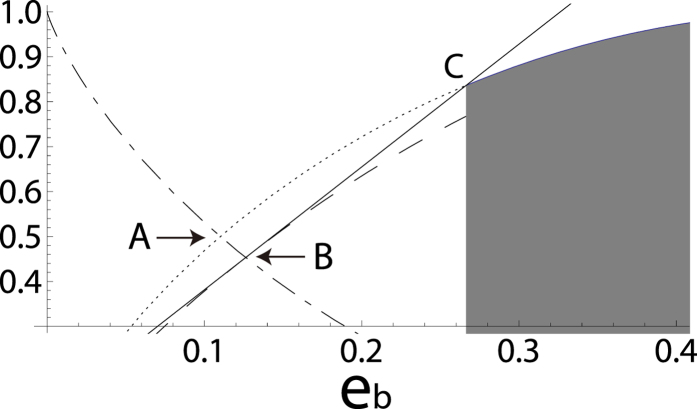
Plot of *H*(*Z*|*X*)^(1,2)^ (dashed line), *h*(*e*_b_) (dotted line), 1 − *h*(*e*_b_) (dot-dashed line), and a tangent (solid line) whose tangent point (**B** = (0.12619.., 0.54690..)) is the intersection of 1 − *h*(*e*_b_) and *H*(*Z*|*X*)^(1,2)^. We can neglect any point in the gray-filled regime in the maximization of 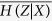.

Here, 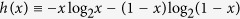, and 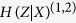 is depicted in Fig. 1 as the dashed line, in which *h*(*e*_b_) (dotted line), 1 − *h*(*e*_b_) (dot-dashed line), and a tangent (solid line) are also plotted. The bit error rate of the intersection (**A**) of the dotted line and the dot-dashed line represents the bit error rate threshold of BB84, and the one (**B**) of the dot-dashed line and the dashed line represents the bit error rate threshold of the six-state protocol up to *N* = 2. As for **C**, it is the intersection of the dotted line and the tangent whose tangent point is **B**.

In maximizing *H*_av_(*Z*|*X*), we have to consider taking the convex combination of *H*(*Z*|*X*)^(1,2)^ and *H*(*Z*|*X*)^(*N*)^ for *N* ≥ 3. Recalling that *H*(*Z*|*X*)^(*N*)^ for any *N* ≥ 3 can never be larger than *h*(*e*_b_) (dotted line) as we use the squash operator for BB84, the convex combination involves only a point along (or under) the dashed line and another point along (or under) the dotted line. This means in particular that we can neglect any point in the gray-filled regime (recall that **C** is the intersection of the dotted line and the tangent whose tangent point is **B**). This is so because adopting a point within the gray-filled regime as the counter-point of the point along the dashed line only decreases the gradient of the line, which is irrelevant for the maximization. Fortunately, according to a simple analysis involving the symmetry of rotations, it turns out that the minimum bit error rate is strictly larger than 25.677 … % for *N* ≥ 4, which is the minimum bit error rate of the gray-filled regime. Therefore, we are left with working only on *N* = 3 case.

#### Analysis for *N* = 3

For the derivation of *H*(*Z*|*X*)^(3)^, we first consider symmetrization of the state 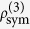 that Alice and Bob share. Recall that our protocol is invariant under the interchange of the basis and bit-flip in each basis. This symmetrization process is represented by a group *G* that is generated by {*R*_*α*_} where *R*_*α*_ is *π*/2 rotation along the *α* basis (*α* = *X, Y, Z*) of a qubit state. Also note that any rotation of the state on 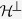, which is an orthogonal complement to 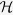 being spanned by 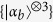, does not change the measurement outcomes since the state on 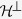 always induces double-click (one can also check this with POVM to be mentioned). Thus, we are allowed to work on the symmetrized density matrix 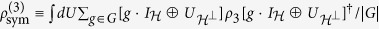. A bit tedious calculation with Schur’s lemma gives us 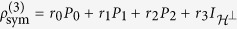, where *r*_*m*_ ≥ 0 (*m* = 0, 1, 2, 3), and *P*_0,1,2_ is a projector onto the subspace spanned by 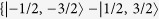, 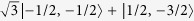, 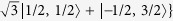, 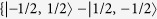, 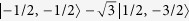, 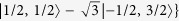 and 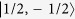, 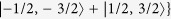. Here, the first (second) index in each ket represents *Z* component of Alice’s (Bob’s) 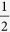-spin (3 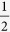-spins with total angular momentum being 3/2) with eigen values being 1/2 and −1/2 (3/2, 1/2, −1/2, and −3/2).

#### The upper bound of Eve’s information

**Figure 2:**
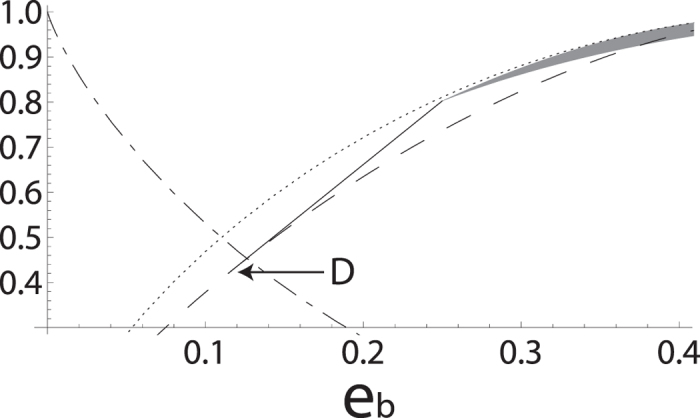
Fig. 1 without the tangent and the one with (*e*_*b*_, *H*(*Z*|*X*)^(3)^) that takes values in the shadow regime. 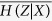
 for 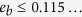 is *H*(*Z*|*X*)^(1,2)^ (dashed line) and the solid line represents 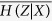 for 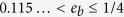. We can obtain 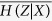 after taking the convex combination of *H*(*Z*|*X*)^(1,2)^ (dashed line) and a point in the shadow regime. Note that as a result of taking the convex combination, the tangent of the dashed line whose tangent point is **B** slightly tilts up, moving the tangent point from **B** to **D** = (0.115 …, 0.42407 …). This leads to the slight degradation in the bit error rate threshold.

To calculate the mutual information, we consider what error rate 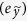 we would have obtained if we had performed the measurement along the 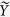 basis onto Alice and Bob’s qubit, in which Bob’s qubit is defined through the BB84 squash operator. Bob’s POVM 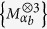 corresponding to detection of the bit value *b* = −1, 1 along *α* basis is represented by 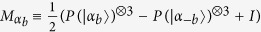, where 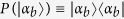 and *I*/2 represents the random assignment of the double-click event, and POVM for detecting the *α*-basis error Γ_*α*_ is 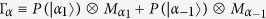. POVM for detecting the 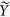-basis error on *qubit pair* is given by 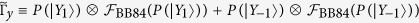, where 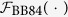 is a map from the qubit space to 3-photon space, which is represented by Kraus operator for the BB84 squash^8^,^9^,^10^. Using all of them, the bit error rate *e*_*b*_ and 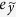 are respectively represented by 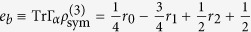 and 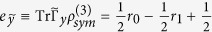, and what we have to do is to derive 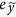 as a function of *e*_*b*_ and to maximize 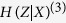. In the equation of *e*_*b*_ and 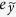, we erase the parameter *r*_3_ by using the condition 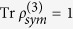, which follows that the positivity condition of 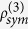 reads *r*_0_, *r*_1_, *r*_2_ ≥ 0 and 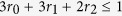. By introducing a parameter set {*t, s, u*} with 0 ≤ *t, u* ≤ 1 and −1 ≤ *s* ≤ 1, we can express *r*_0_ = *ut*(1 + *s*)/6, *r*_1_ = *ut*(1 − *s*)/6, and *r*_2_ = *u*(1 − *t*)/2, and we use this parameterization to derive the regime 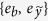 that 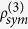 can take. The regime is represented by the triangle with vertices being {1/4, 1/3}, {7/12, 2/3}, and {3/4, 1/2} in 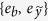-plane, which means that 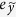 is always bounded by linear functions of *e*_*b*_. This triangle can be translated into the shadow regime in Fig. 2 via 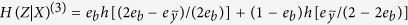 that coincides with 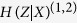 when 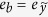, and we note that the tangent in Fig. 1 crosses the shadow regime in Fig. 2 so that the bit error rate threshold should degrade. By considering the convex hull of *H*(*Z*|*X*)^(*N*)^ for *N* = 1, 2, 3, the upper bound of *H*_av_(*Z*|*X*), which we express as 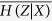, is given by


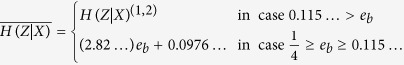


This is also shown in Fig. 2. From this expression, we can derive the bit error rate threshold of 12.6112 … % by solving 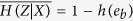 with respect to *e*_*b*_.

#### Remarks

For the first sight, our analysis assumes that Alice and Bob’s pair states are identically and independently distributed. A way to treat unconditional security is to use the argument based on quantum de Finetti theorem^20^ or Azuma’s inequality^21^,^22^,^23^. In the latter argument, we consider an arbitrary whole Alice and Bob’s state, not just a pair state, and we consider to perform the Bell basis measurement from the first qubit pair in order. *ρ*_sym_ is now interpreted as the state of a particular qubit pair conditional on arbitrary Bell basis measurement outcomes. It follows that 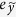 and *e*_*b*_ are probabilities also being conditional on the outcomes, which is required in applying Azuma’s inequality, and most importantly the relation between them are linear as we have already mentioned (for 4 ≤ *N* case, it is given by 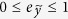 and 0.25677 … ≤ *e*_*b*_). Thus, we can convert our analysis into the analysis of the unconditional security proof by using exactly the same argument as is done in previous papers^22^,^23^.

## Discussion

We prove the unconditional security of the six-state protocol with threshold detectors. For the proof, we propose a technique to determine which photon number states are important, and we employ the squash operator for BB84 and the estimation of the mutual information that can be obtained via the *Y*-basis fictitious measurement on the resulting qubit state. In this paper we consider one-way quantum communication protocol, and our analysis may apply to two-way quantum communication protocol such as BBM92 type QKD^24^, which we leave for the future study. Security proofs of other protocols with threshold detectors are also another future works.

## Additional Information

**How to cite this article**: Kato, G. and Tamaki, K. Security of six-state quantum key distribution protocol with threshold detectors. *Sci. Rep.* **6**, 30044; doi: 10.1038/srep30044 (2016).
